# A tagged visual analog scale is a reliable method to assess keel bone deviations in laying hens from radiographs

**DOI:** 10.3389/fvets.2022.937119

**Published:** 2022-08-18

**Authors:** Lisa Jung, Christina Rufener, Stefanie Petow

**Affiliations:** ^1^Animal Breeding Section, University of Kassel, Kassel, Germany; ^2^Center for Animal Welfare, Department of Animal Science, University of California, Davis, Davis, CA, United States; ^3^Federal Food Safety and Veterinary Office, Bern, Switzerland; ^4^Friedrich-Loeffler-Institut, Institute of Animal Welfare and Animal Husbandry, Celle, Germany

**Keywords:** keel bone fracture, keel bone deviation, assessment method, inter observer reliability, laying hen health

## Abstract

Laying hens often suffer from keel bone damage (KBD) that includes pathologies with different etiologies, like diverse forms of fractures and deviations. Since KBD is a problem in all countries and housing systems, methods for the assessment of deviations are urgently needed. Comparisons between genetic lines and between studies are important to detect underlying mechanisms. Field researchers often use palpation as a low-cost and feasible technique for the assessment of KBD. In contrast to palpation, radiography is effective and highly precise at least in detecting keel bone fractures. The aim of this study was to: i) develop a scoring system to assess keel bone deviations from radiographs, ii) to assess inter- and intra-observer reliability of this scoring system, and iii) to investigate whether fractures and deviations of the keel are correlated. In total, 192 hens were used for the investigation. Digital radiographs were taken and evaluated for all hens after slaughter. We developed a tagged visual analog scale with two extreme images as anchors and four intermediate tags, resulting in six images representing the range from “no deviation” to “highly deviated” on a 10 cm line. Eleven participants scored 50 radiographs of keels with varying degree of severity, whereas five images were scored twice to assess intra-observer reliability. Intraclass correlation coefficient for inter-observer reliability was 0.979 with a confidence interval of 0.968 < ICC < 0.987 (F_49,268_ = 54.2, *p* < 0.0001). Intraclass correlation coefficient for intra-observer reliability was 0.831 with a confidence interval of 0.727 < ICC < 0.898 (F_54,55_ = 10.8, *p* < 0.0001). Individual intra-observer reliability ranged from 0.6 to 0.949. The Spearman correlation showed a strong positive correlation of fractures and deviations (*s*_roh_= 0.803, *p* < 0.001). The tagged visual analog scale could be a reliable instrument for the scoring of keel bone deviations. Our results support the assumption that the majority of highly deviated keels suffer from fractures as well. Further research is needed to investigate the correlation of palpation scores with the evaluation on radiographs.

## Introduction

The keel bone status of commercially kept laying hens is known to be affected by multiple influencing factors, often leading to pathological changes like fractures and deviations. These damages can lead to decreased bird welfare due to pain or immobility (1–6) and economic losses (7, 8). Therefore, the reliability and validity of keel bone damage (KBD) assessment is an intensively discussed topic in laying hen welfare research (9, 10). In 2021, even the European ministers for agriculture discussed the issue of KBD in laying hens, and political support to mitigate the problem can be expected at least in Germany. Hence, it is crucial to provide valid and reliable approaches to capture different forms of KBD.

The keel bone, an extension of the sternum, has a key function in the skeletal system. It is a single large bone on the ventral surface of the body and runs axially along the midline extending outward, perpendicular to the plane of the ribs. The keel provides a large surface where the muscles used for wing motion, the *pectoralis minor* and *pectoralis major* are anchored. Additionally, it protects the inner organs such as liver and heart. At around 16–20 weeks of age hens become sexually mature and begin producing eggs. Due to endogenous calcium resorption for egg shell formation, structural bone content decreases as the laying cycle continuous, resulting in a progressive weakening of bones (11) and thus, increased susceptibility for bone fractures and deviations (12).

Whereas, trauma is assumed to be one main cause for keel bone fractures, deviations could be caused by prolonged pressure on the keel, e.g., due to perching on hard perches (13–16). Due to these differences in etiology, Casey-Trott et al. (17) suggested to assess fractures and deviations as mutually exclusive variables. However, a variety of KBD assessment protocols exist, many of which assess damage as a combination of fractures and deviations (18).

Field researchers often use palpation as a low-cost and feasible technique for the detection of callus material, dislocations, or sharp bends indicating fractures as well as for the detection of deviations from a straight axis of the sternum of live hens. In contrast to palpation, radiography is effective and highly precise in detecting keel bone fractures (19–21). For instance, Rufener et al. (22) developed a reliable method to assess keel bone fracture severity from radiographs, and Baur et al. (21) evaluated the morphology and development of fractures longitudinally. Tracy et al. (23) calculated specificity and sensitivity of radiography based on the true prevalence defined by the visual assessment of dissected keel bones and found that deviations were identified by radiography with a precision of 82.4%. To quantify the severity of a deviation, Eusemann et al. (20) calculated the proportion of deviated keel bone area relative to the area of the whole keel bone from radiographs. As this approach is highly time-consuming, a more efficient though valid and reliable method to assess keel bone deviation severity is needed.

In all, several studies showed benefits of radiography over other techniques to study keel bone fractures as they allow longitudinal on-farm observations in combination with the opportunity for detailed assessment of fracture severity similarly or better than visual inspection after dissection (21, 22). Nevertheless, radiographs might not be sufficient for deviation scoring, as the latero-lateral view of the keel bone might not depict deviations from the sagittal plane appropriately (17, 22). The aim of this study was to: i) develop a scoring system to assess keel bone deviations from radiographs, ii) to assess inter- and intra-observer reliability of this scoring system, and iii) to investigate whether fractures and deviations of the keel are correlated.

## Materials and methods

### Data collection and radiographs

In total, 192 hens were used for the investigation, whereby 102 animals were commercial Lohmann Brown laying hens at end of lay that were selected at the slaughterhouse and 90 hens were animals housed at the Friedrich-Loeffler-Institute (FLI) in Germany, Celle. Out of these 90 hens, 18 were Lohmann Selected Leghorn, and the remaining 72 hens were from experimental lines, WLA and R11. All FLI hens were 33 weeks of age, except 3, that were 25 weeks of age.

Digital radiographs were taken and evaluated for all hens after slaughter. According to Eusemann et al. (20) and Eusemann et al. (24), the carcass was placed on its left side on the digital flat panel detector Thales Pixium 2430 EZ Wireless (Thales Electron Devices S.A., Vélizy-Villacoublay, France) to take the radiograph. Lateral radiographs of the keel region were taken with 50.0 kV and 2 mAs using the X-ray apparatus WDT Blueline 1040 HF (Wirtschaftsgenossenschaft Deutscher Tierärzte eG, Garbsen, Germany) and the X-ray suitcase Leonardo DR mini (Oehm und Rehbein GmbH, Rostock, Germany).

### Development of the deviation scoring system

According to Rufener et al. (22), we aimed to develop a tagged visual analog scale with two extreme images as anchors and four intermediate tags, resulting in six images representing the range from “no deviation” to “highly deviated” on a 10 cm line. To select the images for the tagged visual analog scale, two persons blindly evaluated all x-ray images three times for the presence of deviations, using a six-point scale, where 0 means no deviation at all and 5 means highly deviated. Images that were assigned to the same score in all six rounds were selected as anchors and intermediate tags of the scoring scheme. The remaining 186 x-rays were than assigned by one of the trained assessors using the whole visual analog scale with every value between 0 and 10 for deviations.

**Figure 1:**
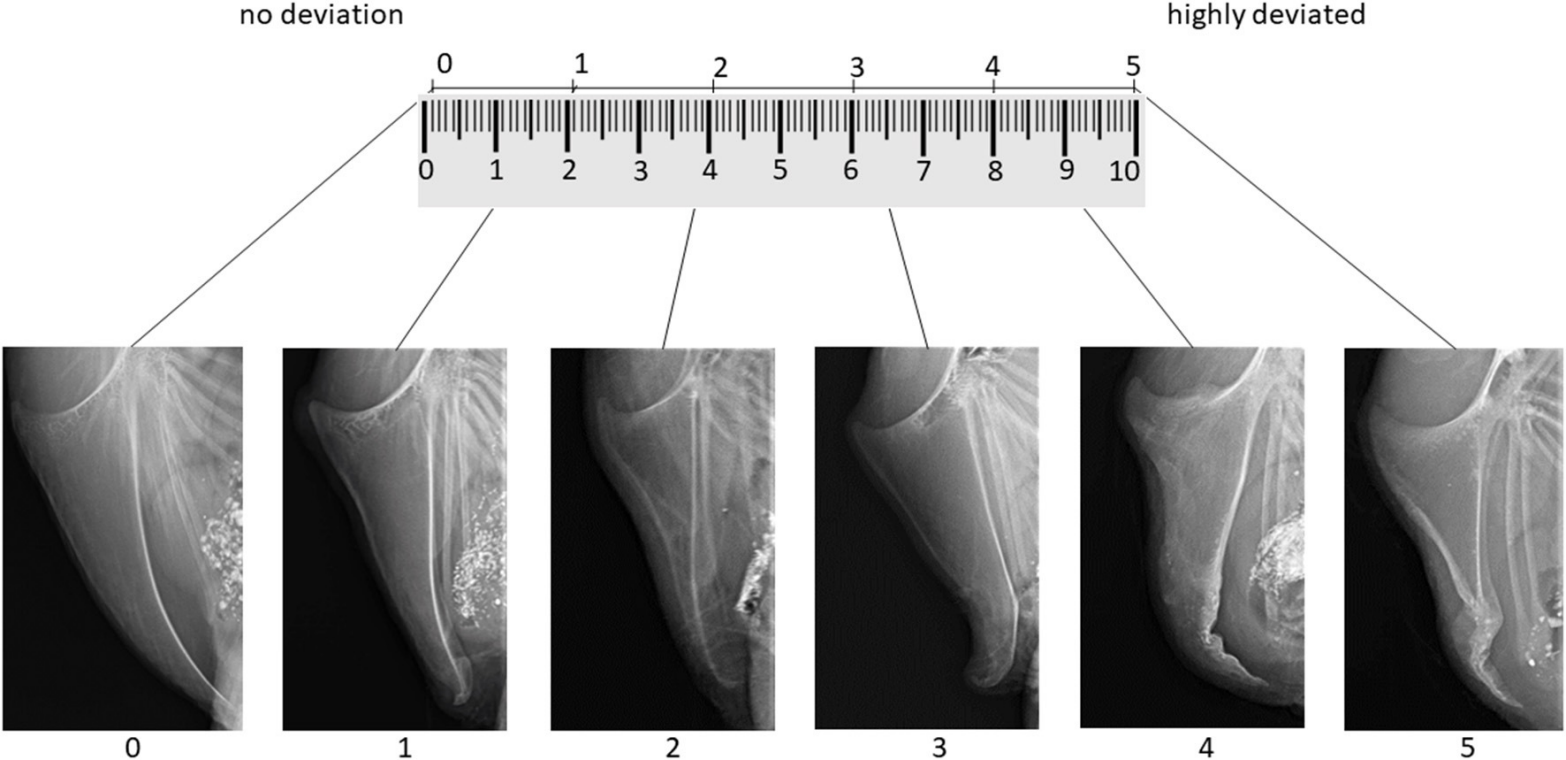
Tagged visual analog scale for the evaluation of keel bone deviations on x-rays including links to get the original x-ray in full size.

As suggested by McCormack et al. (25), the images anchoring the 10 cm line represented the maximal and minimal extreme of the measured dimension: The image for score 0 (left anchor; “no deviation”) showed a fully ossified keel bone without deviations. For score five (right anchor; “highly deviated”), the image of the keel bone with the biggest deviation from the straight axis was selected from the total set of 192 radiographs. Images representing the intermediate scores 1, 2, 3, and 4 were selected based on intermediate amounts of bone affected by deviations while considering the deviation location(s) most frequently observed within the total set of images. Figure 1 shows the tagged visual analog scale with the selected example x-rays. In addition to the tagged visual analog scale and similar to Rufener et al. (22), an additional “catalog” of example scores with 42 radiographs falling within the respective ranges of one score was provided to help fine-tune scoring.

### Inter- and intra-observer reliability test for deviations assessed on radiographs

To assess inter- and intra-observer reliability, we created an e-tutorial providing background information, an introduction to the scoring system, a training session, as well as a scoring session. The e-tutorial is available by contacting the corresponding author or at https://elearning.easygenerator.com/9fecc16a-5cff-4b2b-bc7e-febc1cae8501/. The introduction of the e-learning tool gave a background on the detection of deviations using radiographs, explained the aim of both the scoring system and the reliability trial and gave detailed instructions on the use of a continuous analog scale and the example score catalog. All required documents (scaling tool, example score catalog, and empty scales for scoring) were provided as PDF files. For the training sessions, all 42 images used in the example score catalog were presented in a random order. Users had to select the score range (single choice of “score 0,” “score 1,” “score 2,” “score 3,” “score 4,” or “score 5”) of an image using the scaling tool only and received feedback immediately on whether their response was correct. After completion of the training session, participants of the reliability trial scored 50 images with varying degree of severity. Images were presented on the screen and participants were asked to mark a 10 cm scale on a sheet of paper for each image or to note any possible score between 0.0 and 10.0 in an excel sheet. For the scoring session, participants could use both the scaling tool and the example score catalog. After completion of the scoring session, participants were asked to scan their scoring sheets and send it to the trial coordinator. Distance from the left end of the scale (score 0) to the mark was measured with a ruler and entered into a spreadsheet. Total length of the scale was measured as well in order to correct for distortions (scale = 10 cm), e.g., due to different printer settings. Five images were scored twice to assess intra-observer reliability. In total, 11 persons from different countries and varying experience participated in the online-tutorial and the reliability trial (Table 1).

**Table 1:** Country, background, and experience of participants of the reliability trial for the assessment of keel bone deviations in laying hens on radiographs.

**Background**	**Country**			**Experience with**		
		**Laying hens**	**Keel bones**	**Palpation or dissection**	**Radiographs in general**	**Keel bone radiographs**
Scientist	Germany	Yes	Yes	Yes	Yes	Yes
Scientist	Germany	Yes	Yes	Yes	No	No
Scientist	Germany	Yes	Yes	Yes	No	No
Scientist	Germany	No	No	No	No	No
Scientist	Germany	Yes	Yes	No	No	Yes
Scientist	Switzerland	Yes	Yes	Yes	Yes	Yes
Veterinarian	Germany	No	No	No	Yes	No
Technician	Germany	Yes	Yes	No	Yes	Yes
Technician	Germany	Yes	Yes	Yes	No	Yes
Student	Netherlands	Yes	Yes	No	No	No
Student	Canada	Yes	Yes	No	Yes	Yes

An Intraclass correlation coefficient (ICC) estimate and its 95% confident intervals were calculated using R 3.4.0 (26), package “irr” (27) based on an average-rating (k = 11), absolute-agreement, two-way random-effects model (28) to assess inter-observer reliability. For intra-observer reliability, an ICC estimate and its 95% confident intervals were calculated based on a single-rating, absolute-agreement, two-way mixed-effects model (29, 30). To demonstrate the range of intra-observer reliability within observers, ICCs were additionally calculated for each observer (k = 11) separately. According to the recommendations of Cichetti (31) reliabilities were considered poor (ICC < 0.40), fair (0.40 < ICC < 0.59), good (0.6 < ICC < 0.74), or excellent (0.75 < ICC < 1.0).

### Correlation between deviations and fractures

In addition to the deviation scoring, all radiographs were evaluated for fracture severity by a trained assessor. Using the system of Rufener et al. (22), severity of keel bone fractures was ranging between 0.0 (no fracture) and 10.0 (extremely severe) on a continuous scale. In addition, the number of fractures per radiograph was assessed. Fractures were defined as visible fracture lines or healed fractures with signs of oedema, dislocation, or angulation (21).

Subsequently, a Spearman correlation coefficient was used to calculate the correlation between the severity of fractures and deviations. Additionally, we determined the smallest score for deviations above which a keel was always also affected by fractures. As the keels differed substantially in damage level we conducted all analyses for the whole as well as for the two different data sets slaughterhouse and FLI. All statistics were carried out in R 4.0.2. version 2020 using the packages ggplot2 (32) and tidyverse (33).

## Results

### Inter- and intra- observer reliability of the deviation scoring system

Intraclass correlation coefficient for inter-observer reliability was 0.979 with a confidence interval of 0.968 < ICC < 0.987 (F_49,268_ = 54.2, *p* < 0.0001). Intraclass correlation coefficient for intra-observer reliability was 0.831 with a confidence interval of 0.727 < ICC < 0.898 (F_54,55_ = 10.8, *p* < 0.0001). Individual intra-observer reliability ranged from 0.6 to 0.949.

### Fracture and deviation prevalence from radiographs

**Figure 2:**
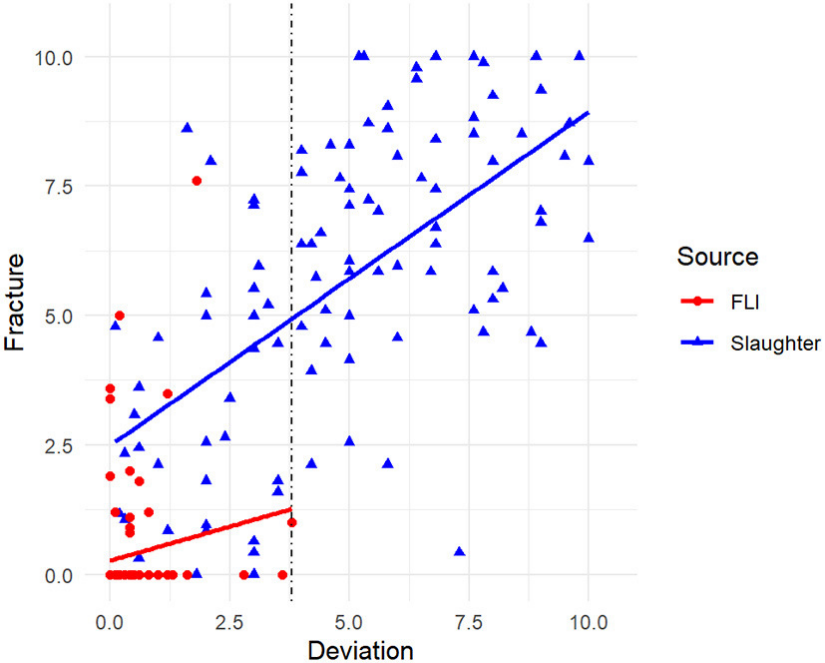
Correlation between deviation and fractures assessed from 192 radiographs of laying hen keel bones sampled at the slaughterhouse (blue) and FLI (red). The vertical dotted line indicates the deviation score threshold of 3.8 from which onwards fractures were always present.

Table 2 shows the range with mean and standard deviation for the whole data set, the keels sampled at the slaughterhouse and the keels sampled at the FLI. Data are summarized in Figure 2.

**Table 2:** Shows minimum, maximum, mean and standard deviation (SD) for all scored radiographs, the slaughterhouse hens, and the FLI hens concerning deviations and fractures.

		**N**	**Minimum**	**Maximum**	**Mean**	**SD**
Full data set	Deviation	192	0	10.00	2.85	3.01
	Fracture	192	0	10.00	3.20	3.46
Slaughterhouse hens	Deviation	102	0.1	10.00	4.96	2.67
	Fracture	102	0	10.00	5.69	2.85
FLI hens	Deviation	90	0	3.80	0.46	0.68
	Fracture	90	0	7.60	0.39	1.18

### Correlation between deviations and fractures

**Figure 3:**
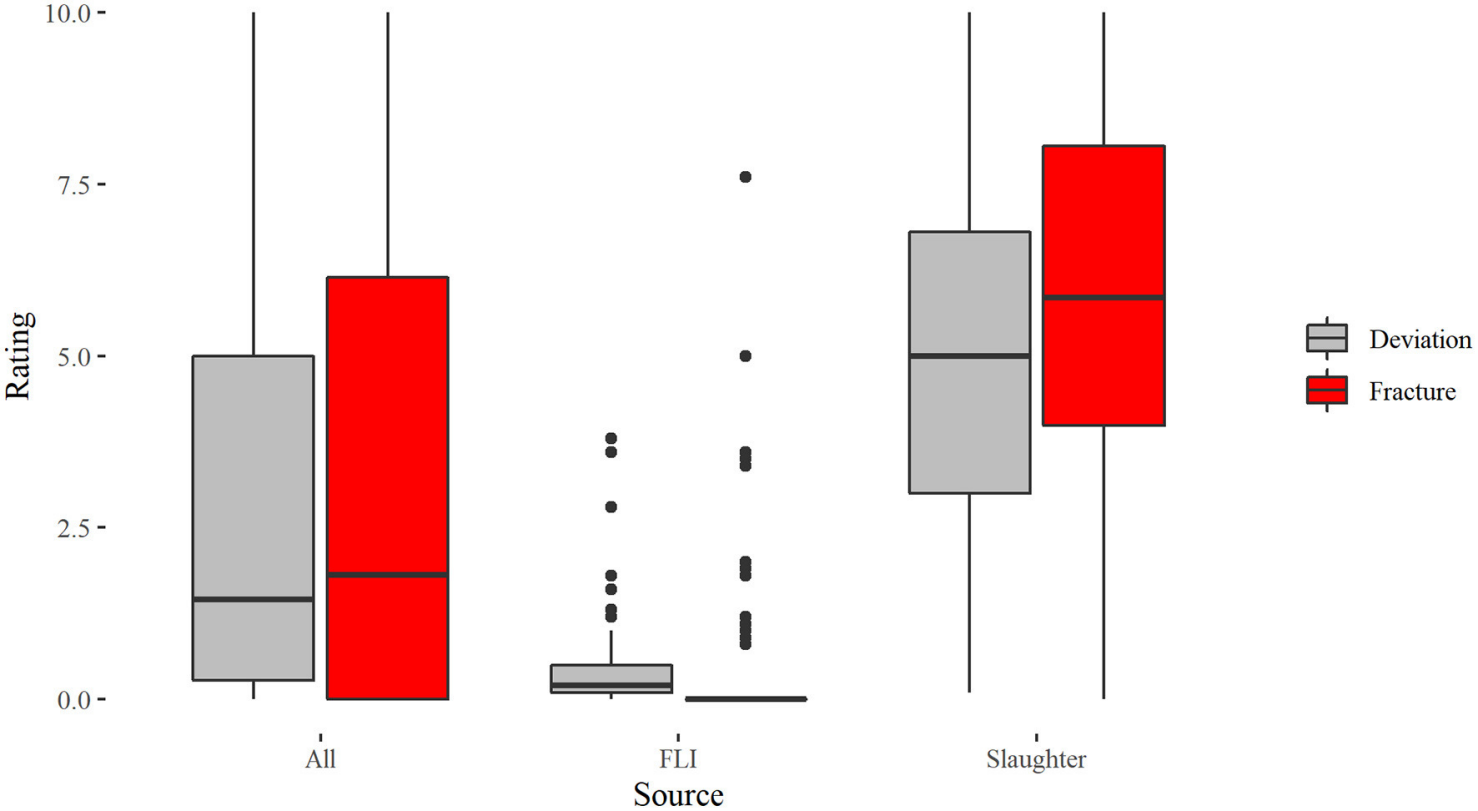
Boxplots for the scoring of deviations and fractures in laying hens assessed on 192 x-rays differentiated according to the total sample “All,” keels sampled from FLI and keels sampled from slaughterhouse hens.

The Spearman correlation showed a positive correlation of fractures and deviations (*s*_roh_= 0.803, *p* < 0.001, *N* = 192). As shown in Figure 3, from a score of 3.8 for deviations onwards, at least one fracture was also present. Scores for fractures of these 66 keels with deviation score ≥ 3.8 were: min: 0.4, max: 10.0, mean: 6.95, median: 7.12.

The Spearman correlation for the slaughterhouse keels showed a positive correlation of fractures and deviations (*s*_roh_= 0.603, *p* < 0.001, *N* = 102), whereas no correlation between fractures and deviations could be found for the FLI keels (*s*_roh_= 0.11, *p* = 0.302, *N* = 90).

Examples for the severity of deviation and fractures evaluated on one laying hen keel bone are given in Table 3. All radiographs are given in Supplementary material A.

**Table 3:** Eighteen randomly selected examples for laying hen keel bone x-rays that show in any case a fracture when the threshold of 3.8 for deviation is exceeded (last six examples).

**ID**	**Severity**		**Difference**
	**Deviation**	**Fracture**	
8738	0	0	0
8818	0	0	0
8778	0	1.9	1.9
92	0.6	2.4	1.8
99	0.6	3.6	3.0
8805	1	0	1
52	1	4.6	3.6
8,731	1.2	3.5	2.3
8804	2.8	0	2.8
75	3	5.5	2.5
47	3	7.1	4.1
8850	3.6	0	3.6
60	4	4.8	0.8
10	4.5	4.5	0
29	6.8	8.7	1.9
50	7.6	10	2.4
26	8	8	0
39	9.8	10	0.2

## Discussion

Without an objective classification of KBD, efforts at linking the causes and effects of different types of keel bone pathologies are severely hindered. For instance, the use of fractures and deviations as distinct traits could be important for selection in breeding, since their etiology and thus, heritability could be different. Given that–to our knowledge–no time-efficient though reliable and valid approach for scoring deviations from radiographs exists, the main objective of this study was to develop and test the reliability of a tagged visual analog assessment scale that provides continuous measurement of deviation severity on radiographs.

The development of the scale was based on the study of Rufener et al. (22), who already achieved excellent reliability for the rating of keel bone fractures with a continuous scale, even though the application of intermediate tags on a visual analog scale is neither common nor recommended due to probable clustering around the tags (34–36). In contradiction, Lansing et al. (37) retain some of the advantages of tagged scales because observers are supported in making consistent choices. Other studies investigating welfare issues in farm animals using a visual analog scale without tags, e.g., for pain measurement in dogs or applying the Welfare Quality^®^ protocol for sheep, reached good to high observer agreements (38, 39). In the study of Nalon et al. (40) inter- and intra-observer reliabilities were higher with a tagged visual analog scale than a 2-point scale (inter-OR: 0.73 v. 0.60; intra-OR: 0.80 v. 0.67). Following the investigation of Rufener et al. (22) where the reliability of a scoring system assessing the aggregate severity of multiple fractures resulted in excellent intra- and inter-observer reliabilities, our results were promising with similar values from good to excellent individual intra-observer values (0.6 to 0.949) and excellent inter-observer values (0.968 to 0.987), indicating a high inter- and intra-participant agreement (31) and minimal measurement error by observers (41). This can be seen as a big improvement compared to achieved IOR values in studies that used discrete 2-, 3- or 4-point scales for palpation (23, 42–45). Breed and age differences may affect the accuracy of the results obtained, especially when comparing the two groups in our study because both deviation and fracture severity differed markedly between slaughterhouse and FLI hens (mean deviations: 0.46 vs. 4.96, mean fractures 0.39 vs. 5.69). Similarly, deviations and fractures were more prevalent in slaughterhouse hens than in FLI hens (deviations: 67.2 vs. 97.9%, fractures: 60.4 vs. 97.4%), though the difference in prevalence was less pronounced compared to the severity of KBD. Keel bone fracture prevalence of the slaughterhouse hens is in agreement with Baur et al. (21) who found that 97.0% of the hens kept in aviaries had at least one fracture. Although it is tempting to conclude that the housing environment (commercial vs. experimental) caused the differences in prevalence and severity of the lesions, our data sets cannot be compared. Instead, the more pronounced difference in severity than prevalence warrants attention and could further inform about underlying mechanisms. For example, almost all slaughterhouse hens (97.9%) and the majority (67.2%) of experimental hens had deviations. Whereas, prevalence of deviations was 1.45 times higher in slaughterhouse hens than in experimental hens, deviation severity was more than 10 times higher in slaughterhouse hens compared to experimental hens (0.46 vs. 4.96). This comparison indicates that individual deviations were not only less frequent, but also of lower severity in experimental hens. Hence, underlying causes for damage might be comparable across housing systems resulting in high prevalence irrespective of the hen's environment (18), though hens' susceptibility for severe damage might vary depending on housing, management, and genetics. In the same vein Thøfner et al. (46), found different morphologies of fractures in cage- and aviary-housed hens, but pathogenesis appeared similar across housing systems. Overall, measuring severity of deviations and fractures in addition to prevalence could increase the validity of KBD research. In addition, our results underline that transferring results from experiments into practice must be done with caution.

Besides the importance of assessing severity of deviations and fractures, the correlation between these pathologies is an important aspect when trying to understand the etiology of KBD. Despite the recommendation of Casey-Trott et al. (17) to assess deviations and fractures as mutually exclusive variables, many publications on keel bone fractures are based on scoring systems looking at overall damage, i.e., fractures and deviations combined (18). One reason for the difficulty in assessing deviations and fractures separately is that the two conditions seem to correlate. In our study, we found that deviations with a severity of ≥ 3.8 were always accompanied by at least one fracture. This result seems in accordance with Scholz et al. (47), who based their scoring system for KBD on histological analyses. In their system, score 1 indicated damage to the keel without fractures, whereas keel bone lesions with scores 2 and 3 showed histological evidence of fractures. Importantly, we cannot conclude whether severe fractures result in deviations, or whether severe deviations are precursors for fractures. Regardless of whether fractures or deviations were there first, the relevance of KBD for animal welfare should be considered. In case of deviations it is not clear to which extent deviated keels with score < 3.8 are relevant to animal welfare. As we found that deviations from score 3.8 onwards are correlated with fractures, we can assume that these are connected with pain or immobility.

A limitation of our study is the lack of verification of deviations scored with a tagged visual analog scale on x-rays in comparison to deviations assessed with a tagged visual analog scale on dissected bones. Tracy et al. (23) calculated specificity and sensitivity of radiography based on the true prevalence defined by the visual assessment of dissected keel bones: deviations were identified by radiography with a precision of 82.4%. A precise identification of the affected keel bone area, e.g., the measurement of the proportion of deviated keel bone area relative to the area of the whole keel bone, could be useful to increase the validity of our proposed scoring system and thus, help to better understand underlying mechanism of KBD. Overall, a tagged visual analog scale is a reliable method to measure the severity of keel bone deviations in laying hens from radiographs. The validity of the method has to be evaluated in further studies, e.g., by comparing deviation severity of dissected bones with deviation severity obtained from radiographs. Assessing the relationship between the severity of keel bone deviations and deviation scoring through palpation could increase practical relevance and improve interpretation of studies where radiography cannot be used for KBD detection.

## Data availability statement

All datasets generated for this study are included in the article/Supplementary material. The raw data supporting the conclusions of this article that are not given in the supplementary material can be requested from the co-authors. Requests to access these datasets should be directed to Lisa Jung, lisa.jung@uni-kassel.de or Stefanie Petow, Stefanie.petow@fli.de.

## Ethics statement

The animal study was reviewed and approved by Lower Saxony State Office for Consumer Protection and Food Safety (LAVES) No. 33.19-42502-04-15/1966.

## Author contributions

LJ prepared the original draft. All authors contributed equally to conceptualization, methodology, formal analysis, investigation, and approved the submitted version.

## Conflict of interest

The authors declare that the research was conducted in the absence of any commercial or financial relationships that could be construed as a potential conflict of interest.

## Publisher's note

All claims expressed in this article are solely those of the authors and do not necessarily represent those of their affiliated organizations, or those of the publisher, the editors and the reviewers. Any product that may be evaluated in this article, or claim that may be made by its manufacturer, is not guaranteed or endorsed by the publisher.
