# Supplementary material for: A tagged visual analog scale is a reliable method to assess keel bone deviations in laying hens from radiographs
Source: Front Vet Sci. 2022 Aug 18;9:937119. doi: 10.3389/fvets.2022.937119 (PMC9434373; doi:10.3389/fvets.2022.937119)
Supplement: Supplementary file 1 [file Data_Sheet_1.PDF]

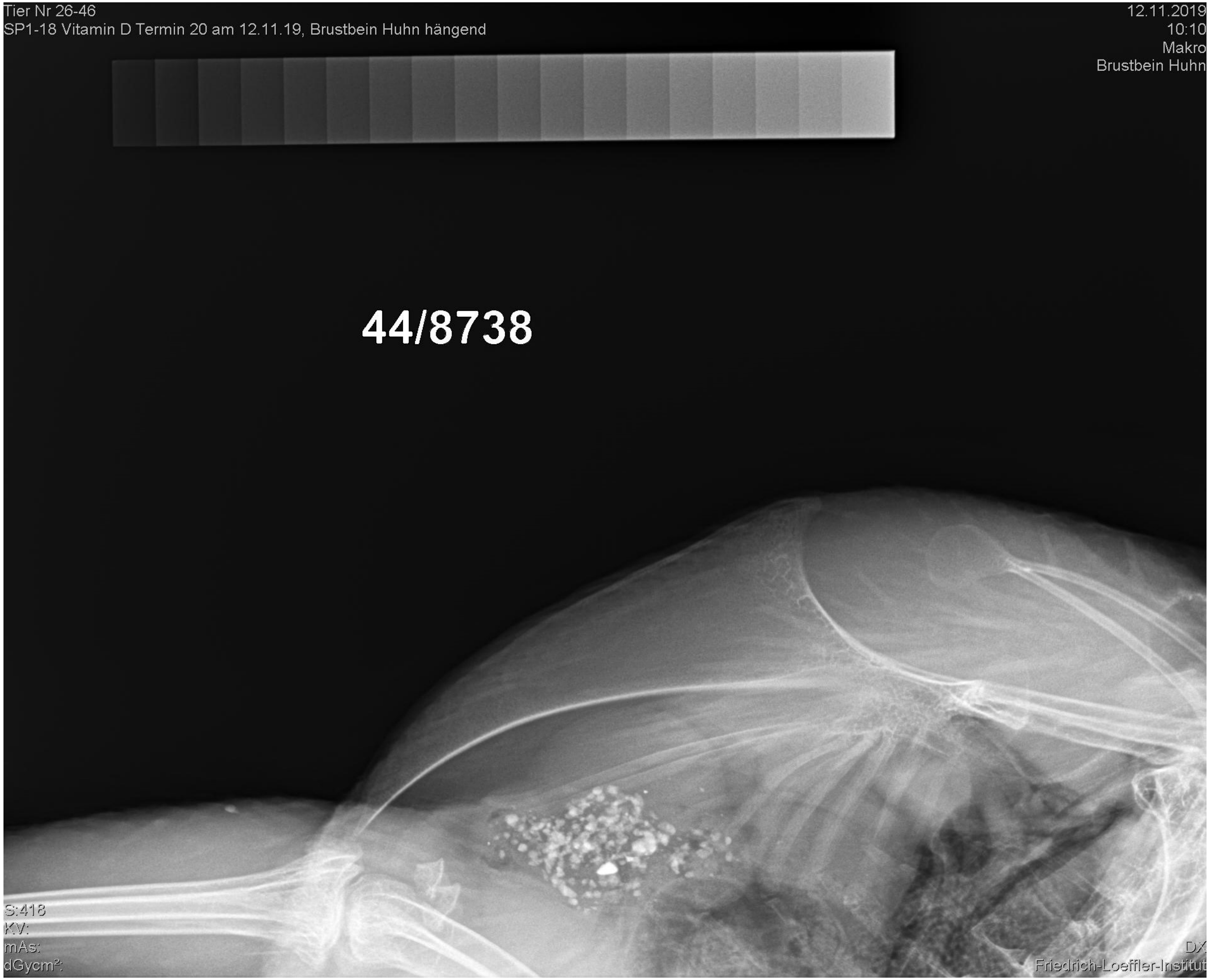
Tier Nr 26-46
SP1-18 Vitamin D Termin 20 am 12.11.19, Brustbein Huhn hängend
12.11.2019
10:10
Makro
Brustbein Huhn
44/8738
S:418
KV:
mAs:
dGycm²:
DX
Friedrich-Loeffler-Institut

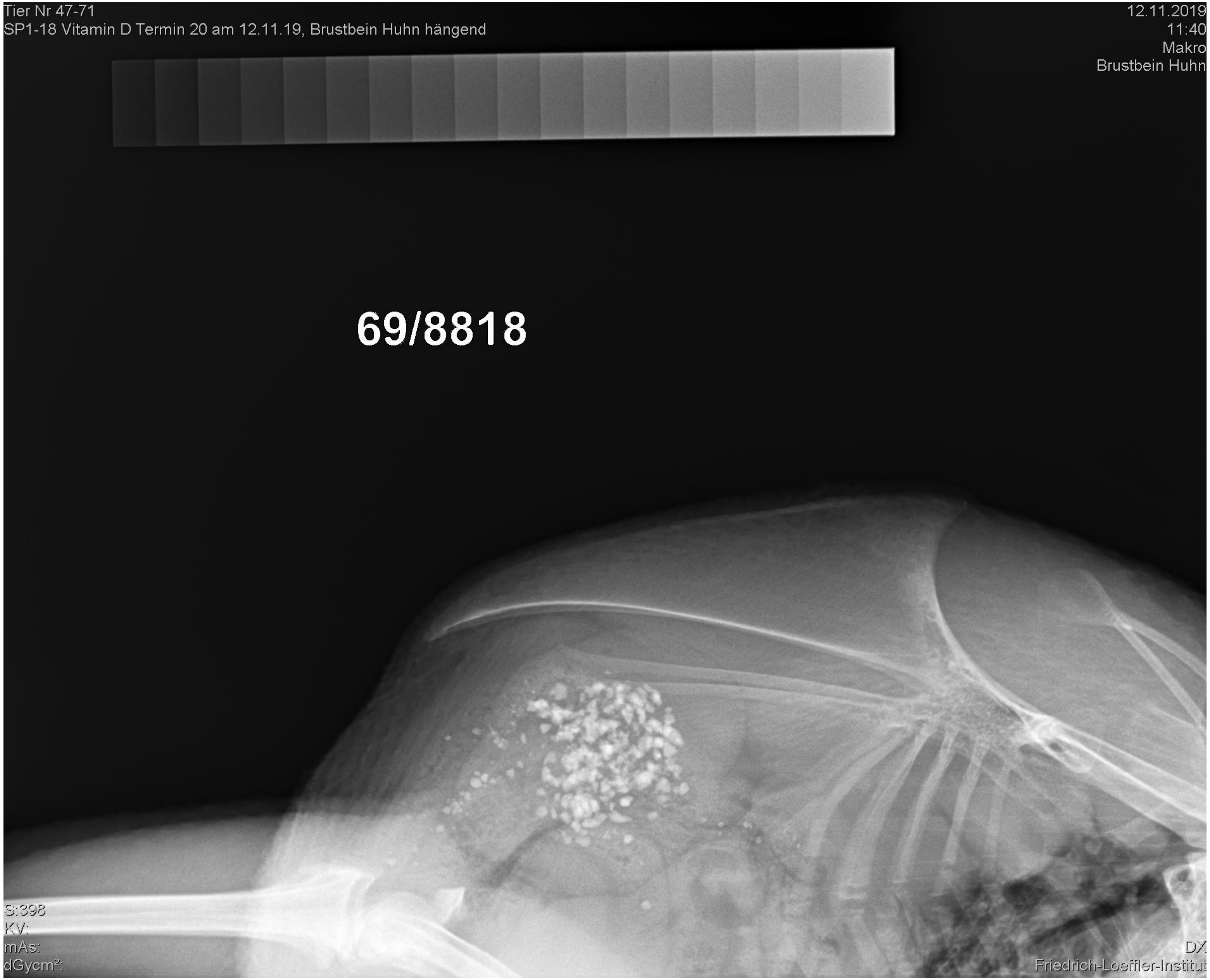

Tier Nr 47-71
SP1-18 Vitamin D Termin 20 am 12.11.19, Brustbein Huhn hängend
12.11.2019
11:40
Makro
Brustbein Huhn
69/8818
S:398
KV:
mAs:
dGycm²:
DX
Friedrich-Loeffler-Institut

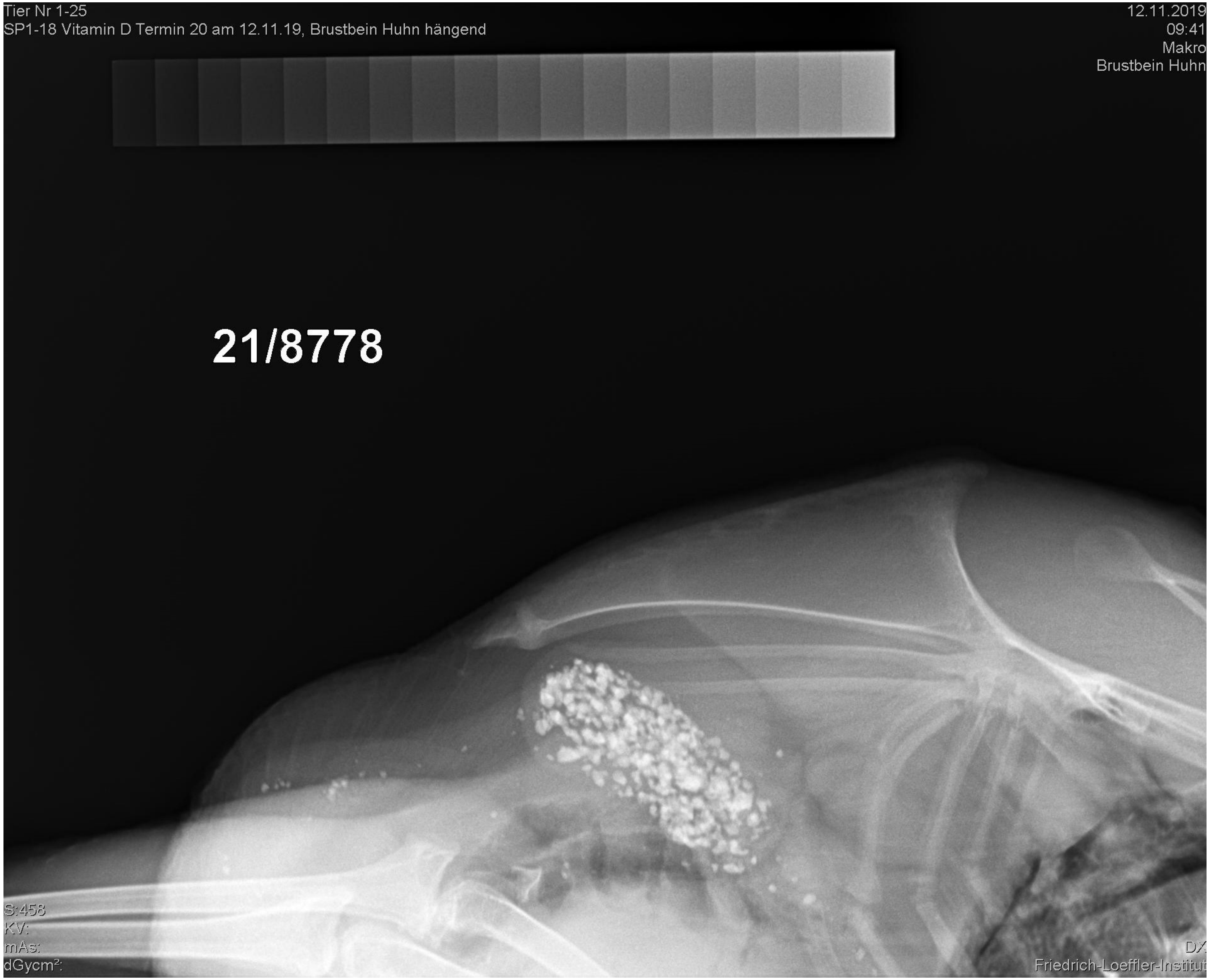
Tier Nr 1-25
SP1-18 Vitamin D Termin 20 am 12.11.19, Brustbein Huhn hängend
12.11.2019
09:41
Makro
Brustbein Huhn
21/8778
S:458
KV:
mAs:
dGycm²:
DX
Friedrich-Loeffler-Institut

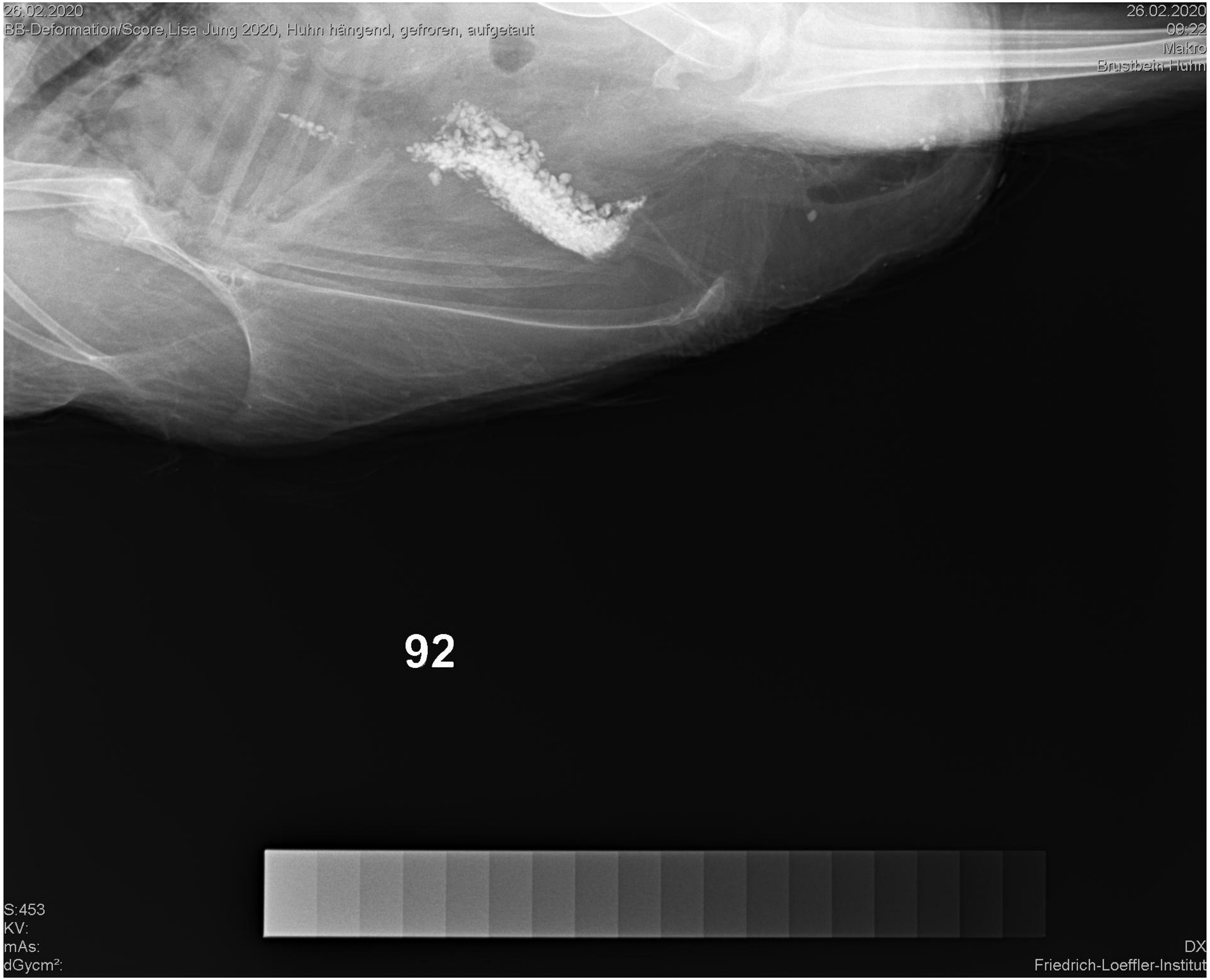
26.02.2020
BB-Deformation/Score,Lisa Jung 2020, Huhn hängend, gefroren, aufgetaut
26.02.2020
09:22
Makro
Brustbein Huhn
92
S:453
KV:
mAs:
dGycm²:
DX
Friedrich-Loeffler-Institut

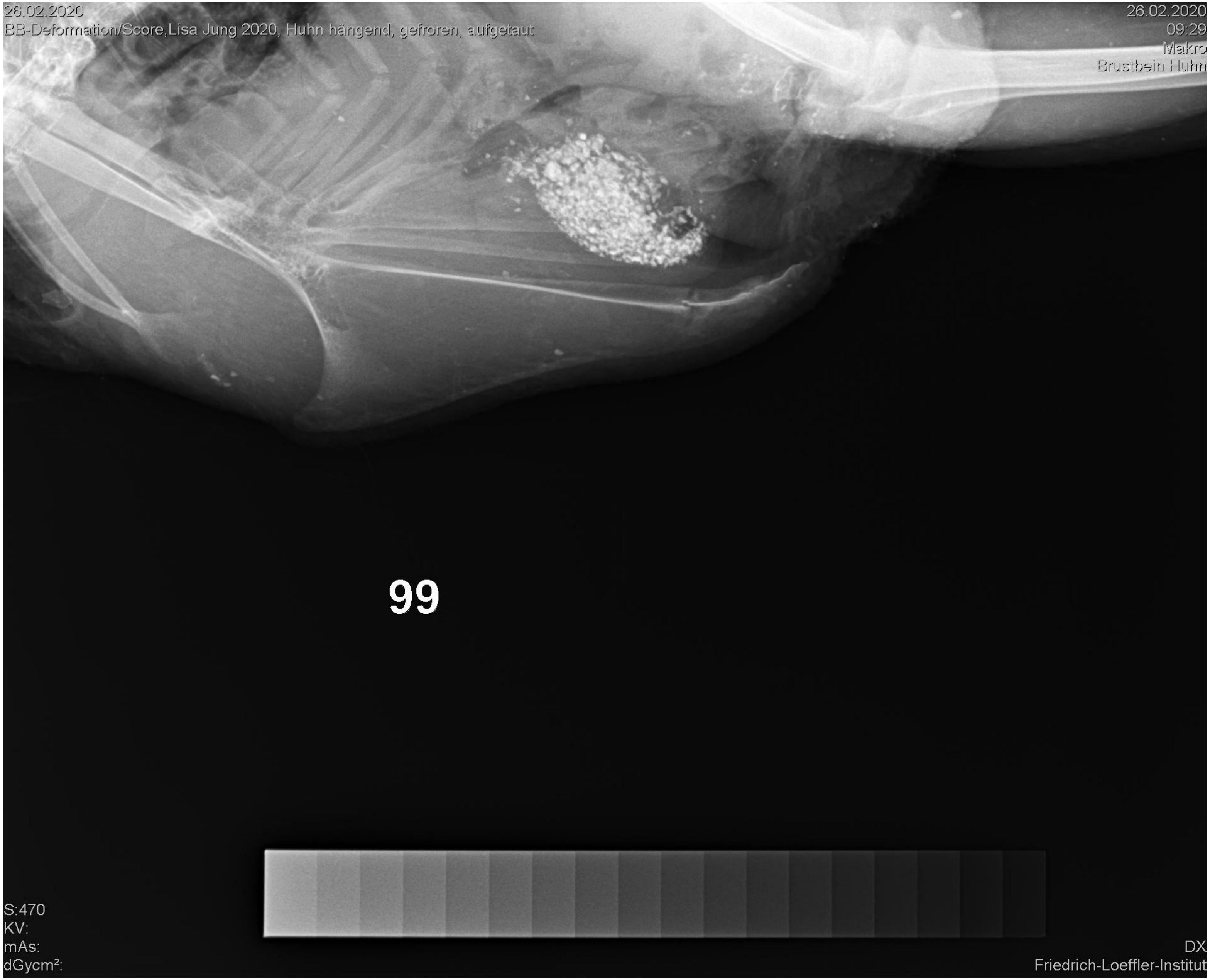
26.02.2020
BB-Deformation/Score,Lisa Jung 2020, Huhn hängend, gefroren, aufgetaut
26.02.2020
09:29
Makro
Brustbein Huhn
99
S:470
KV:
mAs:
dGycm²:
DX
Friedrich-Loeffler-Institut

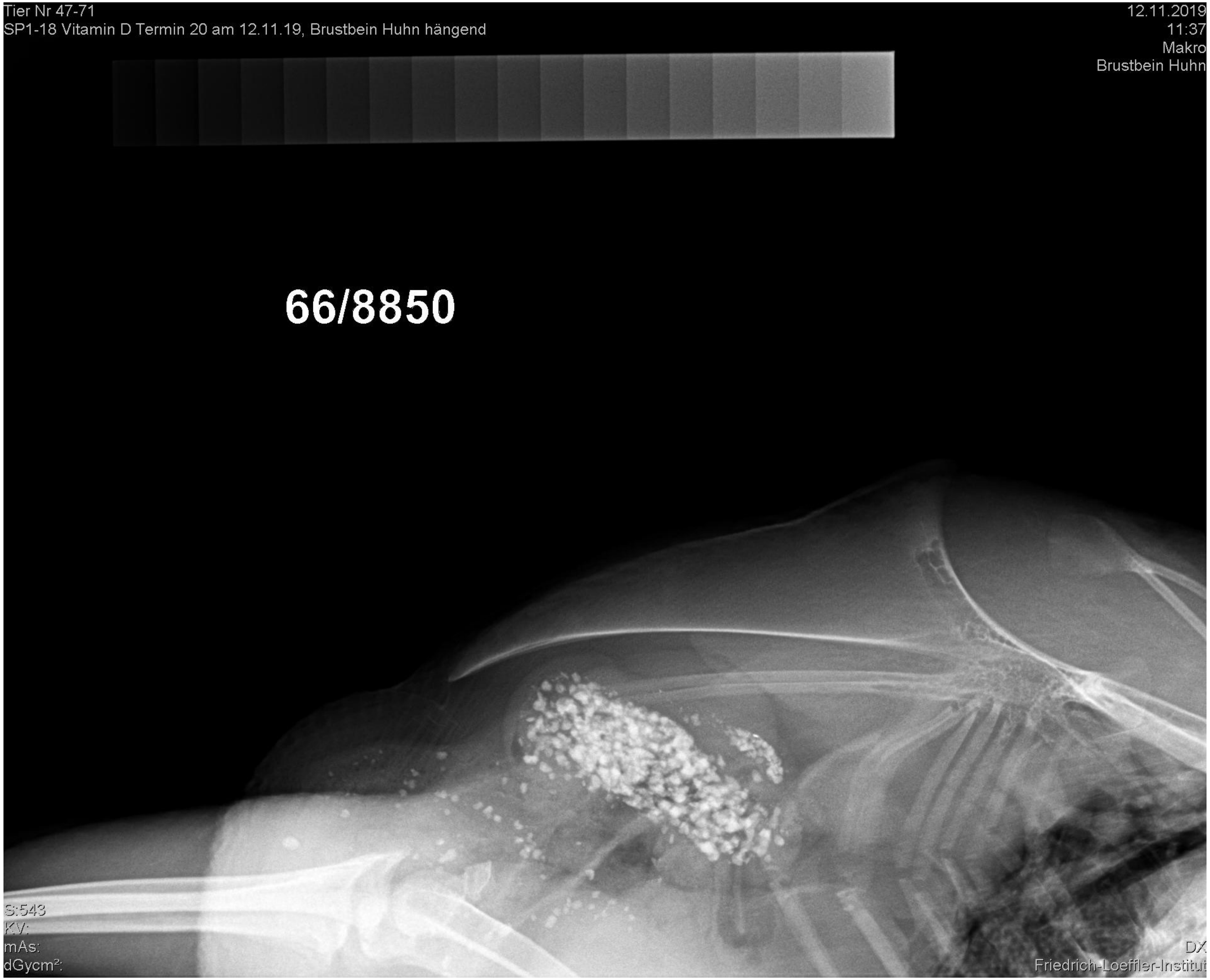
Tier Nr 47-71
SP1-18 Vitamin D Termin 20 am 12.11.19, Brustbein Huhn hängend
12.11.2019
11:37
Makro
Brustbein Huhn
66/8850
S:543
KV:
mAs:
dGycm²:
DX
Friedrich-Loeffler-Institut

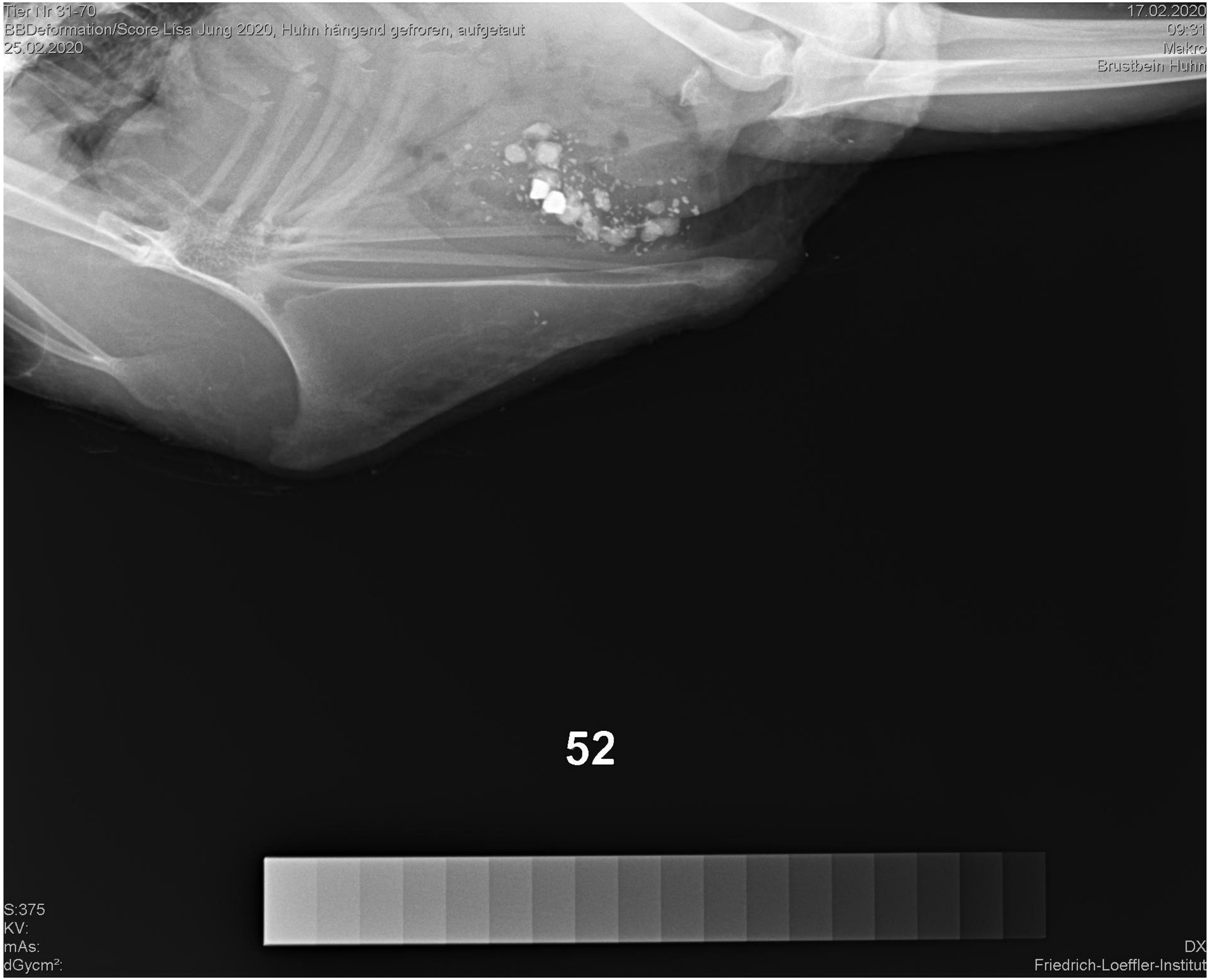
Tier Nr 31-70
BBDeformation/Score Lisa Jung 2020, Huhn hängend gefroren, aufgetaut
25.02.2020
17.02.2020
09:31
Makro
Brustbein Huhn
52
S:375
KV:
mAs:
dGycm²:
DX
Friedrich-Loeffler-Institut

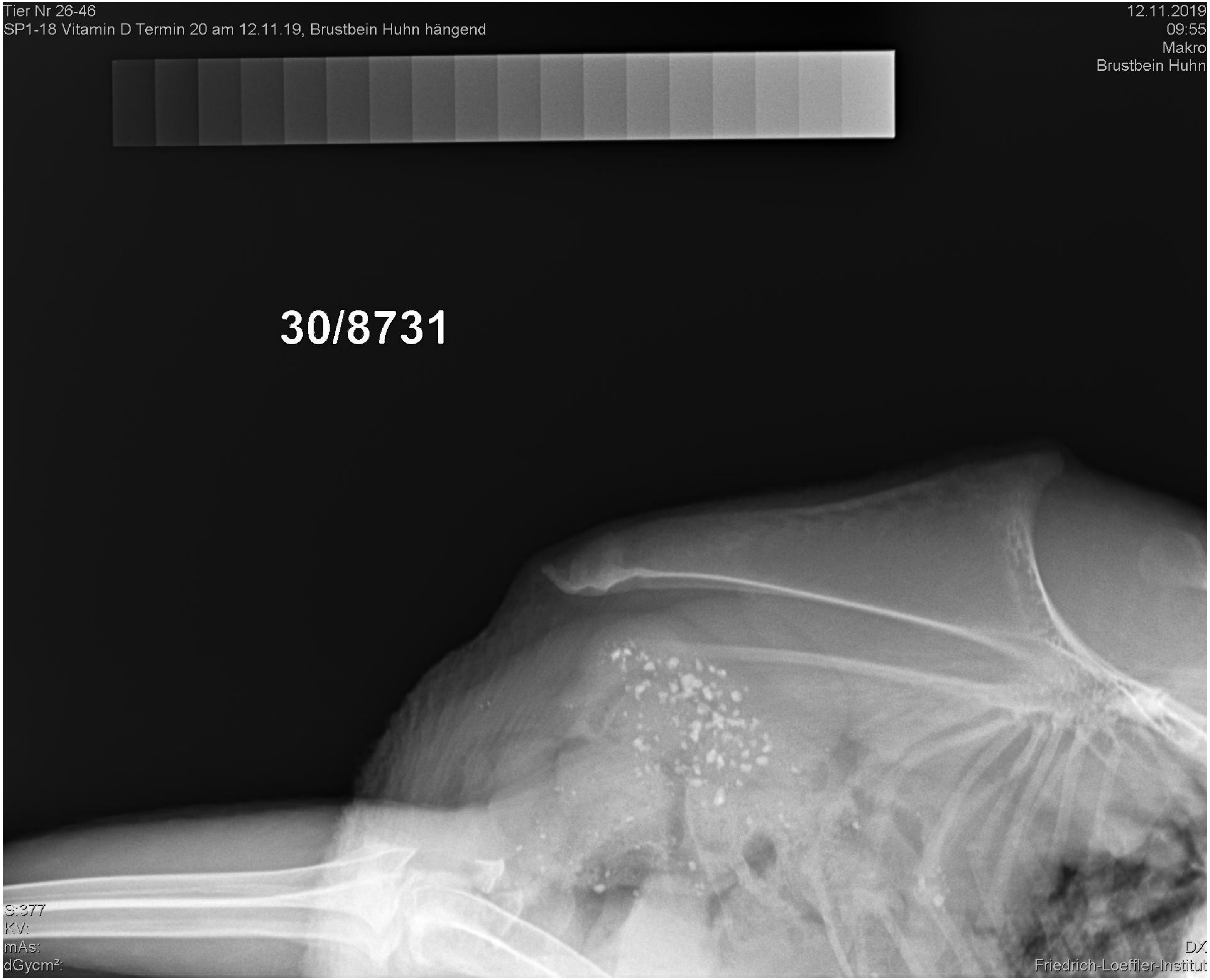
Tier Nr 26-46
SP1-18 Vitamin D Termin 20 am 12.11.19, Brustbein Huhn hängend
12.11.2019
09:55
Makro
Brustbein Huhn
30/8731
S:377
KV:
mAs:
dGycm²:
DX
Friedrich-Loeffler-Institut

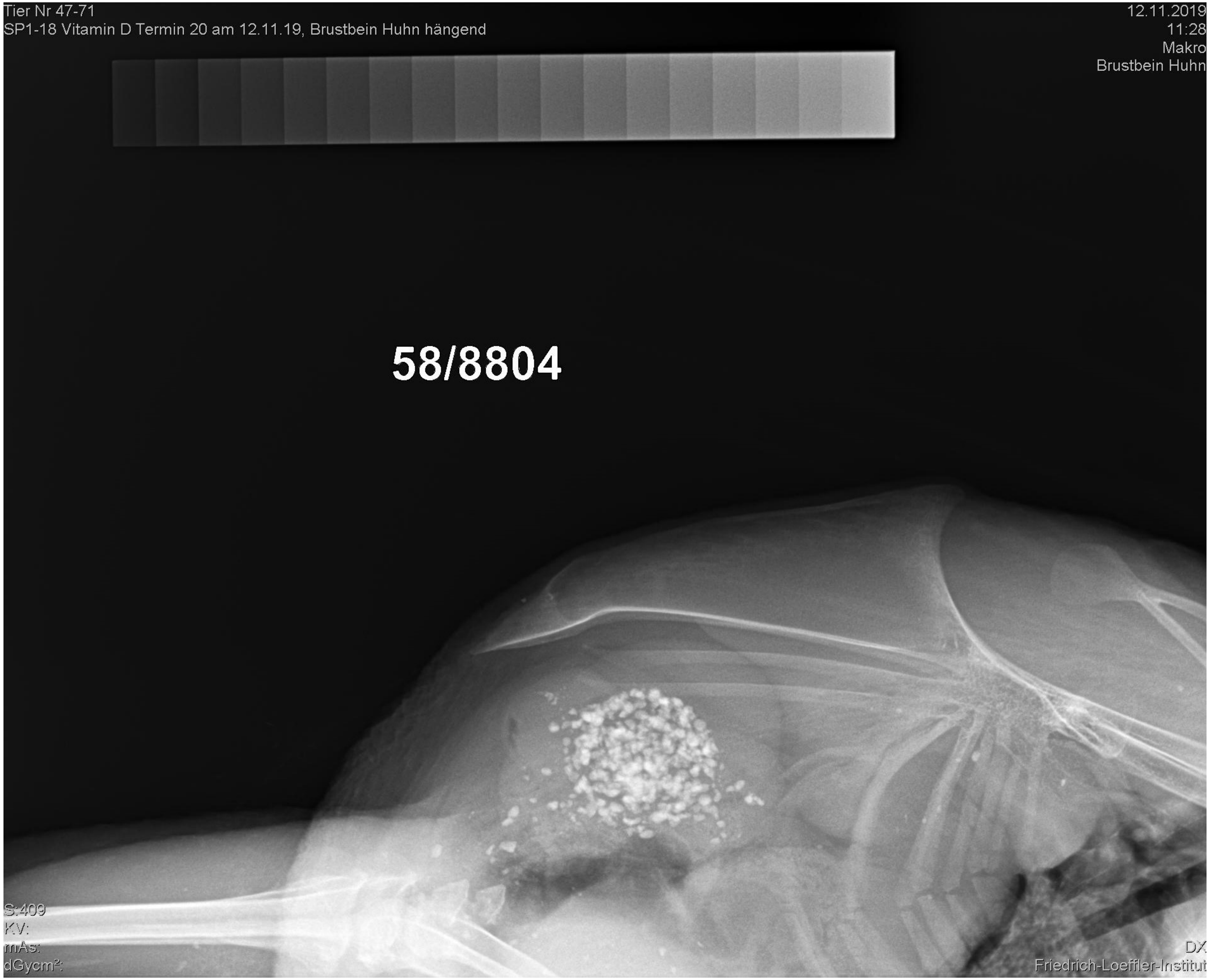
Tier Nr 47-71
SP1-18 Vitamin D Termin 20 am 12.11.19, Brustbein Huhn hängend
12.11.2019
11:28
Makro
Brustbein Huhn
58/8804
S:409
KV:
mAs:
dGycm²:
DX
Friedrich-Loeffler-Institut

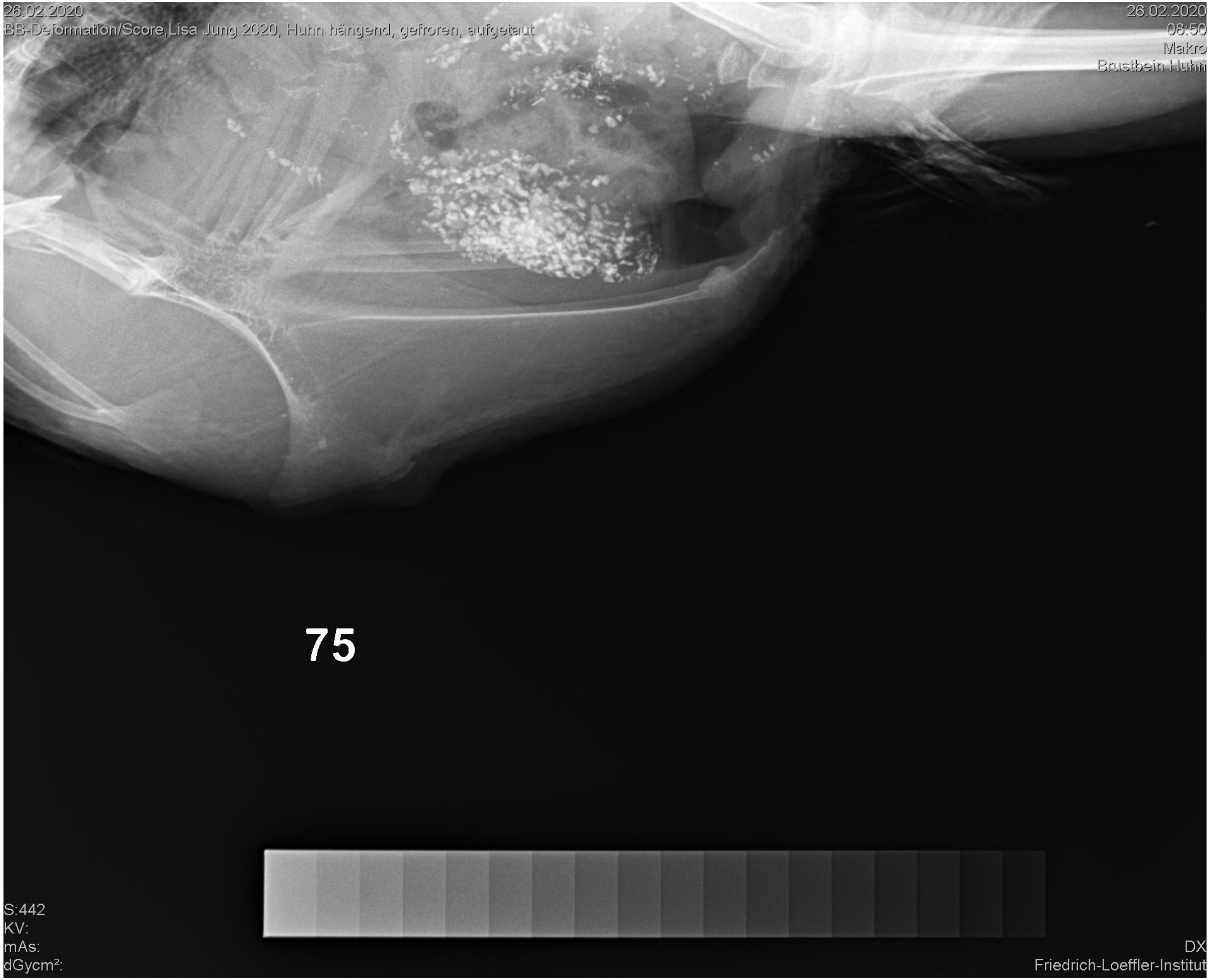
26.02.2020
BB-Deformation/Score,Lisa Jung 2020, Huhn hängend, gefroren, aufgetaut
26.02.2020
08:50
Makro
Brustbein Huhn
75
S:442
KV:
mAs:
dGycm²:
DX
Friedrich-Loeffler-Institut

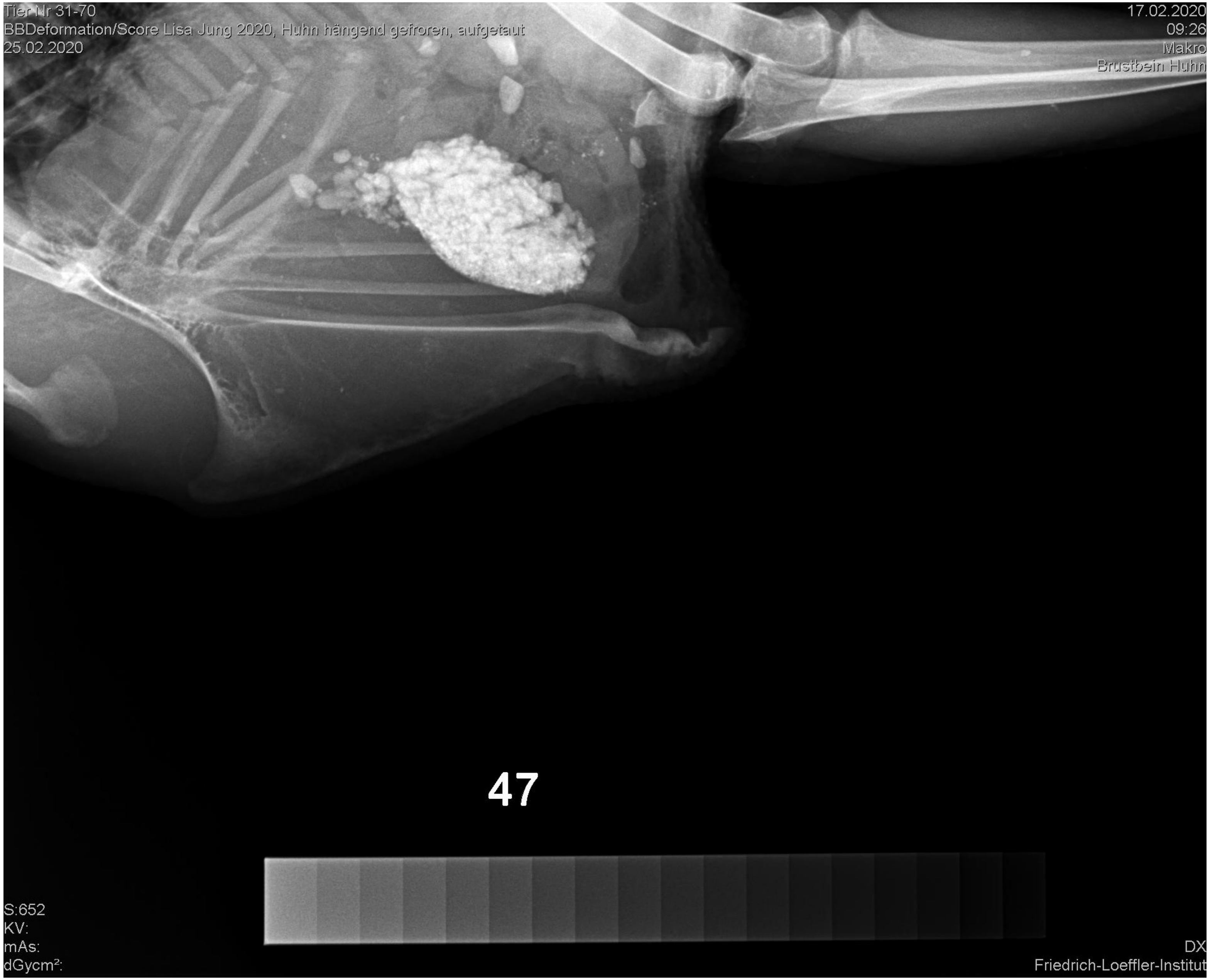
Tier Nr 31-70
BBDeformation/Score Lisa Jung 2020, Huhn hängend gefroren, aufgetaut
25.02.2020
17.02.2020
09:26
Makro
Brustbein Huhn
47
S:652
KV:
mAs:
dGycm²:
DX
Friedrich-Loeffler-Institut

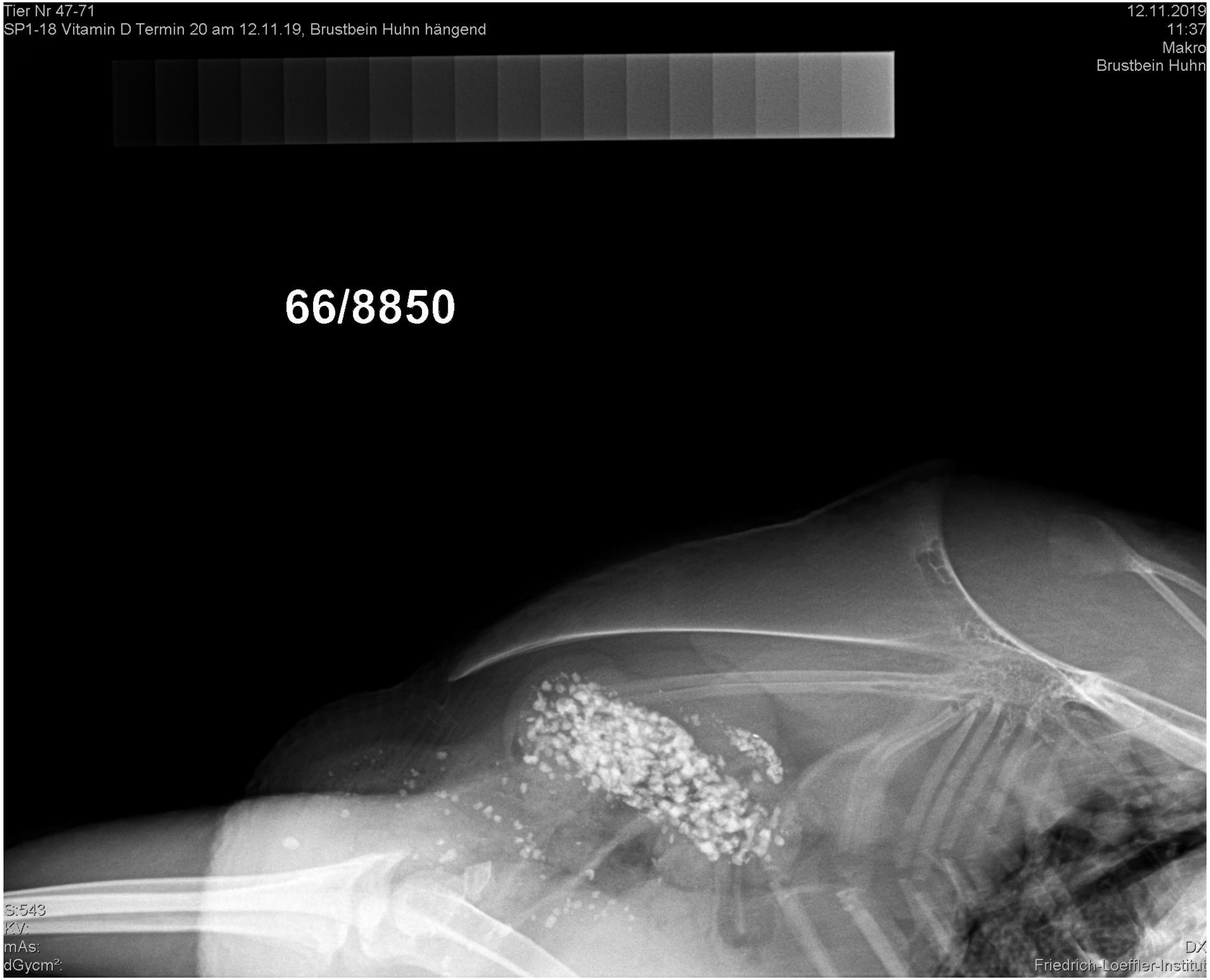
Tier Nr 47-71
SP1-18 Vitamin D Termin 20 am 12.11.19, Brustbein Huhn hängend
12.11.2019
11:37
Makro
Brustbein Huhn
66/8850
S:543
KV:
mAs:
dGycm²:
DX
Friedrich-Loeffler-Institut

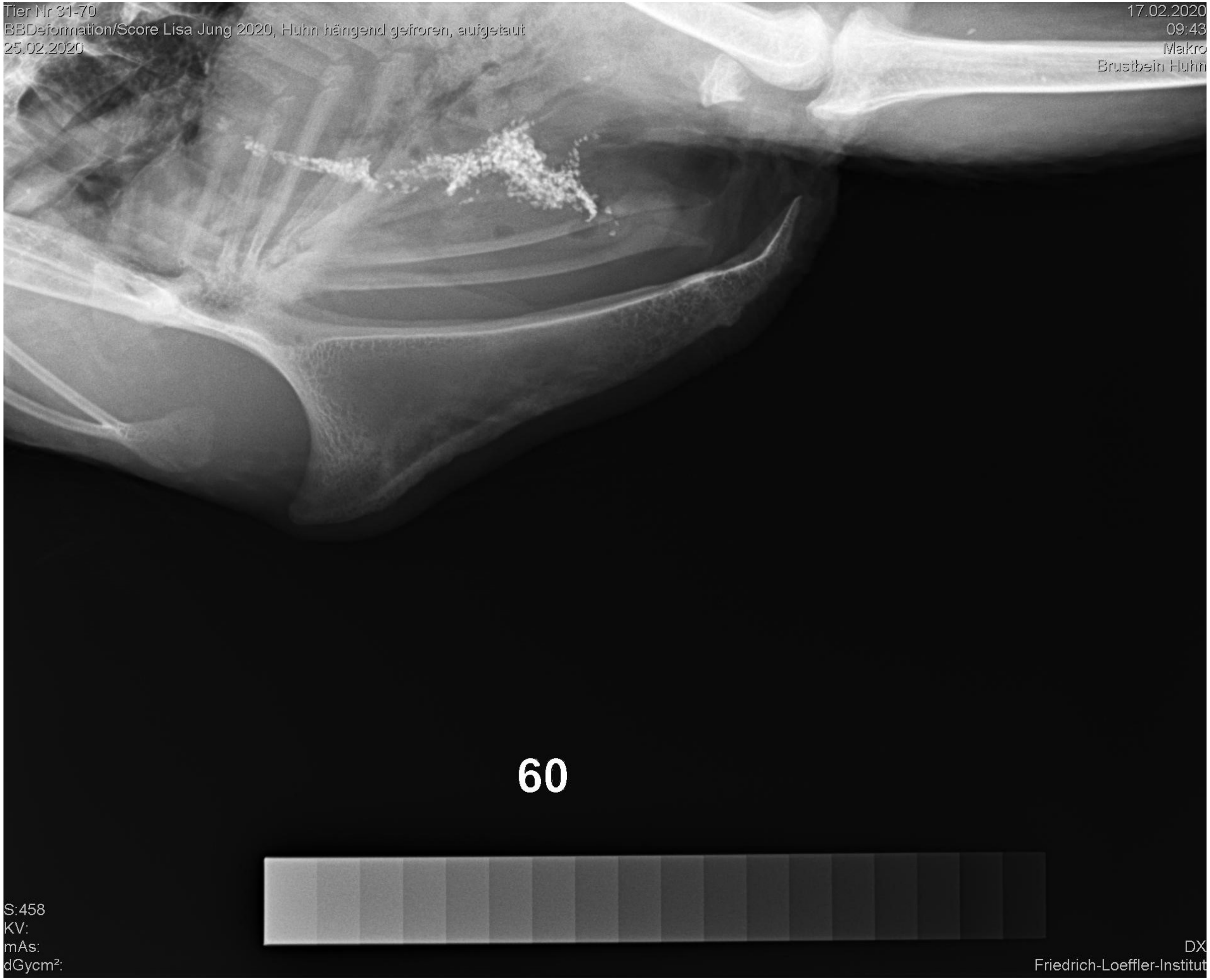
Tier Nr 31-70
BBDeformation/Score Lisa Jung 2020, Huhn hängend gefroren, aufgetaut
25.02.2020
17.02.2020
09:43
Makro
Brustbein Huhn
60
S:458
KV:
mAs:
dGycm²:
DX
Friedrich-Loeffler-Institut

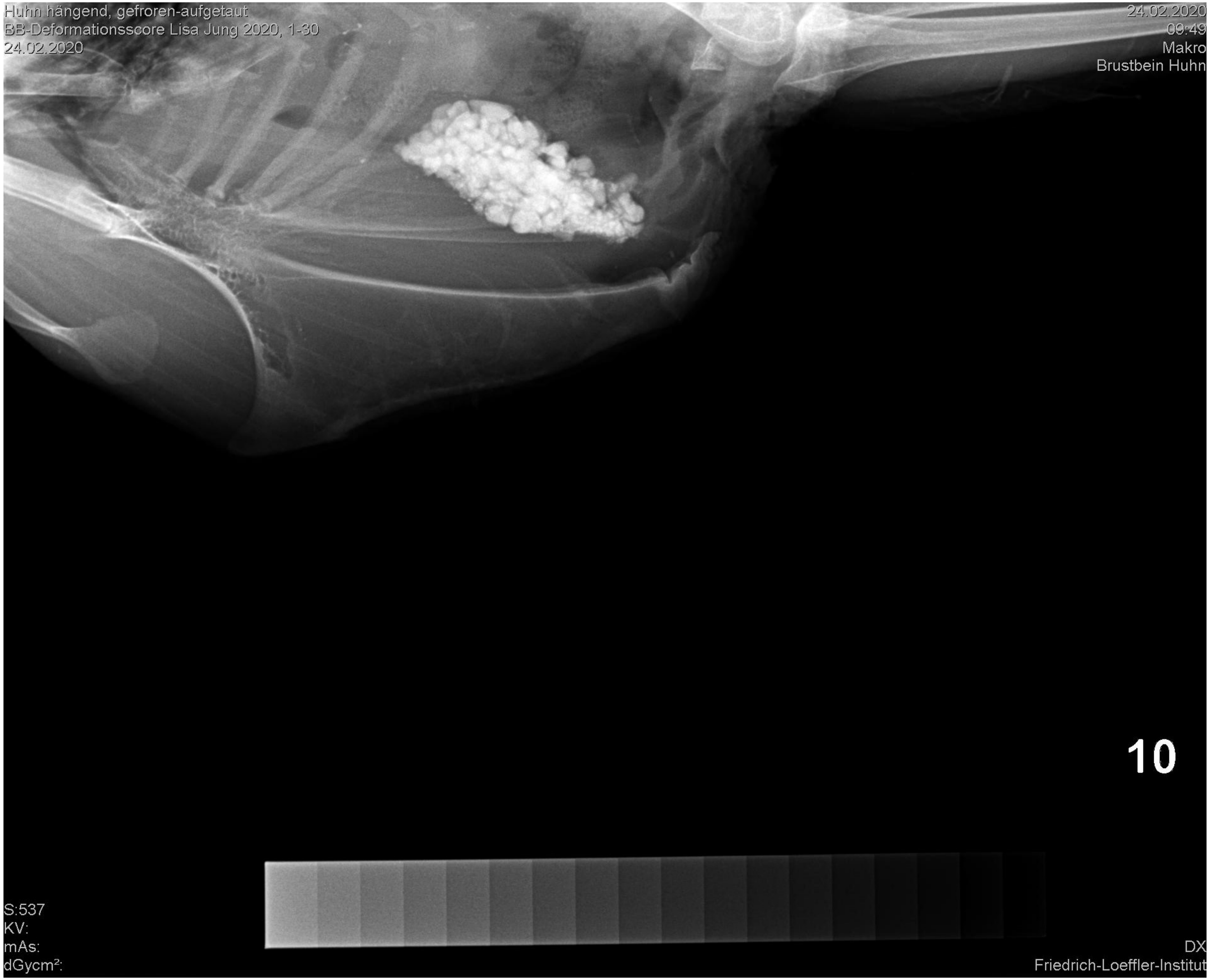
Huhn hängend, gefroren-aufgetaut
BB-Deformationsscore Lisa Jung 2020, 1-30
24.02.2020
24.02.2020
09:49
Makro
Brustbein Huhn
10
S:537
KV:
mAs:
dGycm²:
DX
Friedrich-Loeffler-Institut

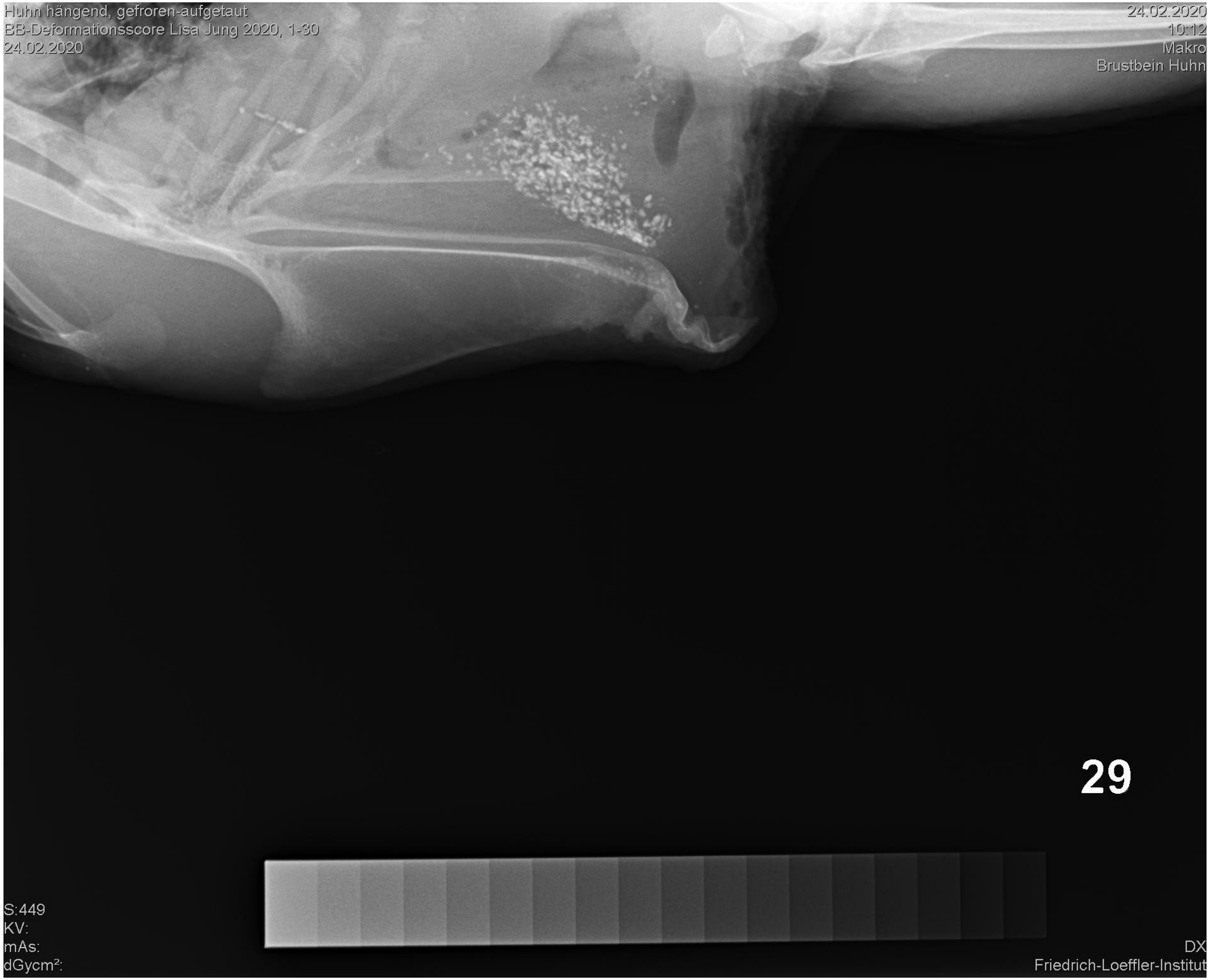
Huhn hängend, gefroren-aufgetaut
BB-Deformationsscore Lisa Jung 2020, 1-30
24.02.2020
24.02.2020
10:12
Makro
Brustbein Huhn
29
S:449
KV:
mAs:
dGycm²:
DX
Friedrich-Loeffler-Institut

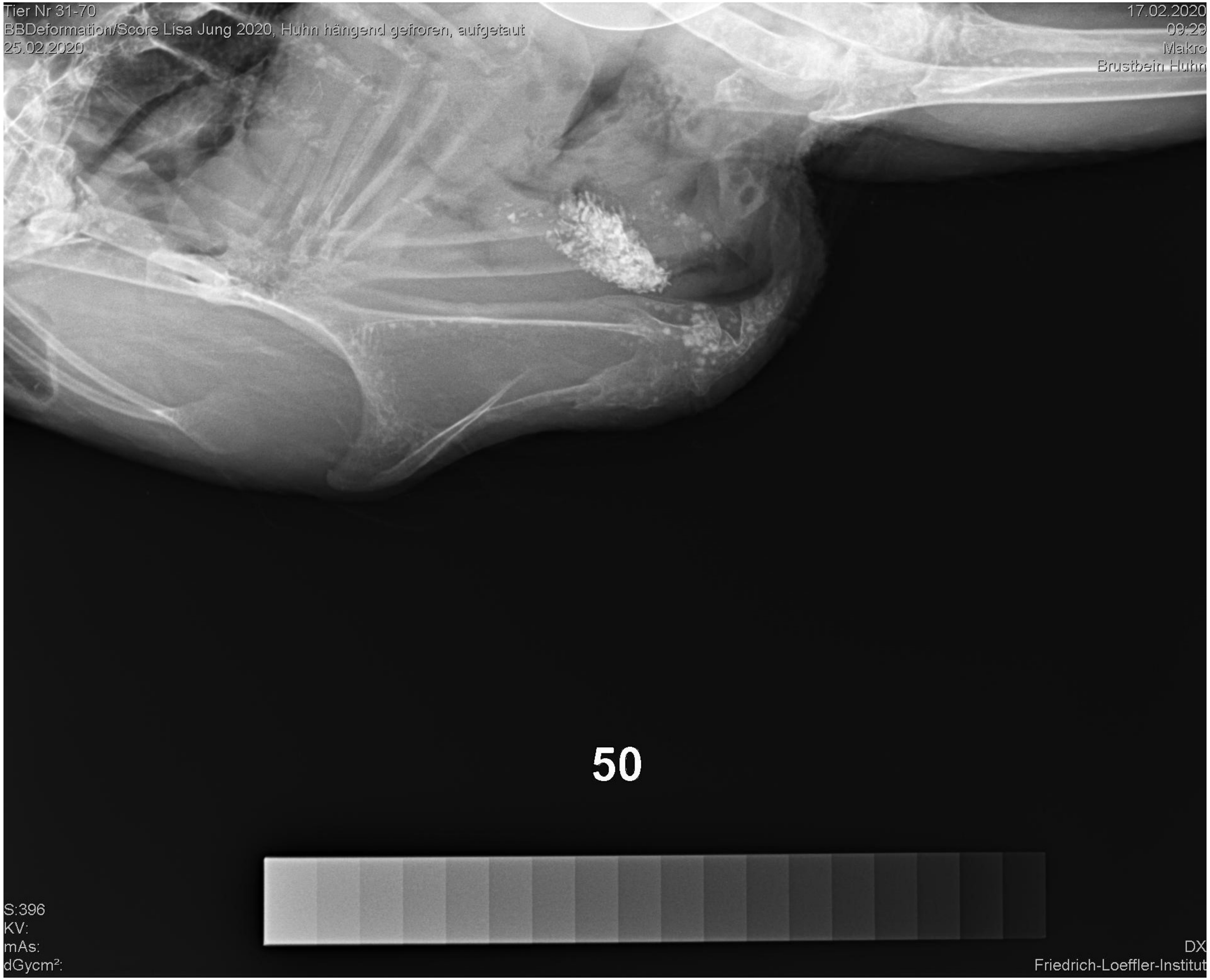
Tier Nr 31-70
BBDeformation/Score Lisa Jung 2020, Huhn hängend gefroren, aufgetaut
25.02.2020
17.02.2020
09:29
Makro
Brustbein Huhn
50
S:396
KV:
mAs:
dGycm²:
DX
Friedrich-Loeffler-Institut

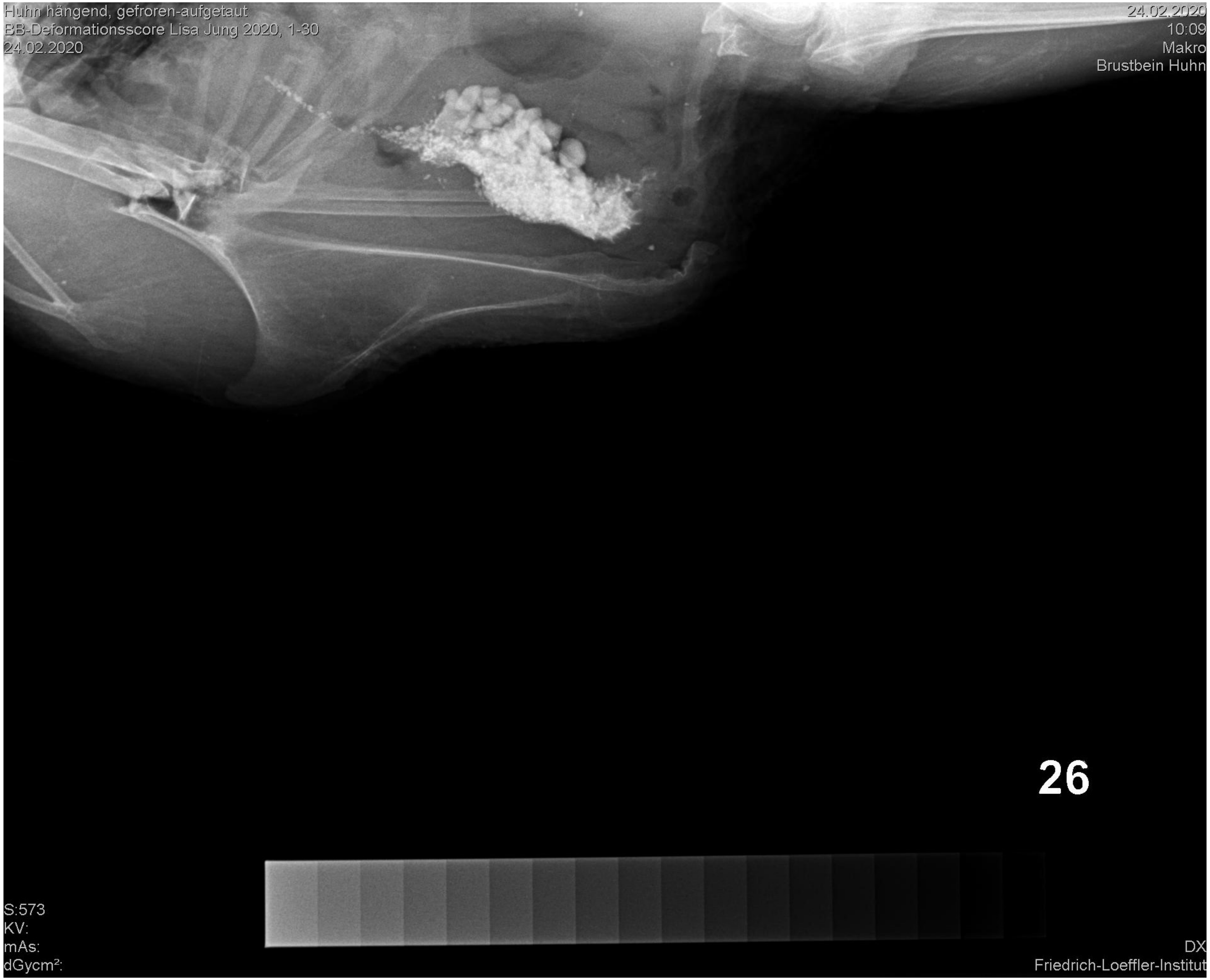
Huhn hängend, gefroren-aufgetaut
BB-Deformationsscore Lisa Jung 2020, 1-30
24.02.2020
24.02.2020
10:09
Makro
Brustbein Huhn
26
S:573
KV:
mAs:
dGycm²:
DX
Friedrich-Loeffler-Institut

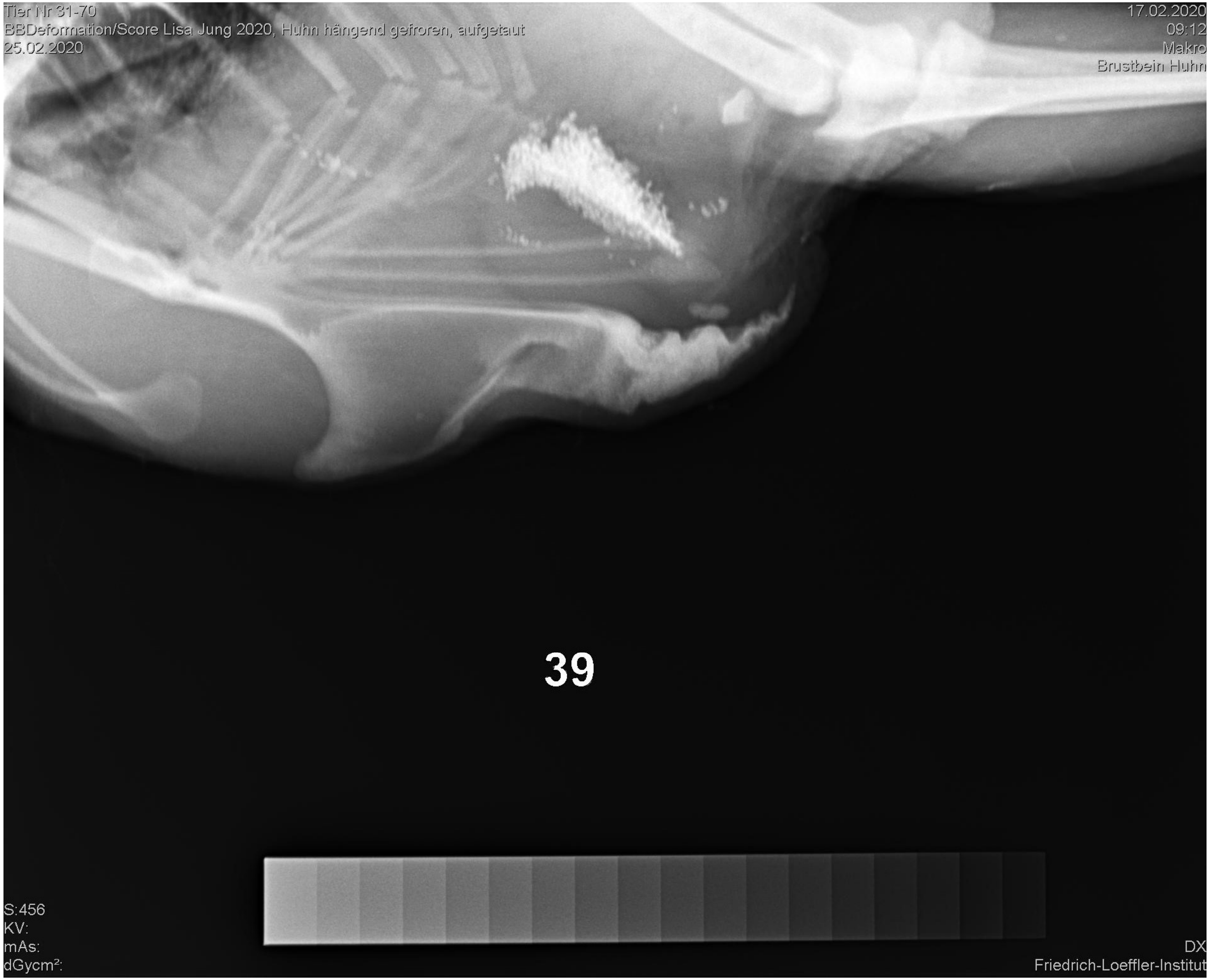
Tier Nr 31-70
BBDeformation/Score Lisa Jung 2020, Huhn hängend gefroren, aufgetaut
25.02.2020
17.02.2020
09:12
Makro
Brustbein Huhn
39
S:456
KV:
mAs:
dGycm²:
DX
Friedrich-Loeffler-Institut
